# Women with disability: the experience of maternity care during pregnancy, labour and birth and the postnatal period

**DOI:** 10.1186/1471-2393-13-174

**Published:** 2013-09-13

**Authors:** Maggie Redshaw, Reem Malouf, Haiyan Gao, Ron Gray

**Affiliations:** 1 Policy Research Unit for maternal Health and Care, National Perinatal Epidemiology Unit, University of Oxford, Old Road, Oxford OX3 7LF, UK

**Keywords:** Disability, Maternity care, Pregnancy, Birth, Maternity survey

## Abstract

**Background:**

It has been estimated that 9.4% of women giving birth in the United Kingdom have one or more limiting longstanding illness which may cause disability, affecting pregnancy, birth and early parenting. No large scale studies on a nationally representative population have been carried out on the maternity experiences of disabled women to our knowledge.

**Method:**

Secondary analysis of data from a survey of women in 2010 by English National Health Service Trusts on behalf of the Care Quality Commission was undertaken. 144 trusts in England took part in the postal survey.

Women self-identified with disability and were excluded if less than 16 years of age or if their baby had died. The 12 page structured questionnaire with sections on antenatal, labour and birth and postnatal care covered access, information, communication and choice. Descriptive and adjusted analyses compared disabled and non-disabled groups. Comparisons were made separately for five disability subgroups: physical disability, sensory impairment, mental health conditions, learning disability and women with more than one type of disability.

**Results:**

Disabled women comprised 6.14% (1,482) of the total sample (24,155) and appeared to use maternity services more than non-disabled women. Most were positive about their care and reported sufficient access and involvement, but were less likely to breastfeed. The experience of women with different types of disability varied: physically disabled women used antenatal and postnatal services more, but had less choice about labour and birth; the experience of those with a sensory impairment differed little from the non-disabled women, but they were more likely to have met staff before labour; women with mental health disabilities also used services more, but were more critical of communication and support; women with a learning disability and those with multiple disabilities were least likely to report a positive experience of maternity care.

**Conclusion:**

This national study describes disabled women’s experiences of pregnancy, child birth and postnatal care in comparison with non-disabled women. While in many areas there were no differences, there was evidence of specific groups appropriately receiving more care. Areas for improvement included infant feeding and better communication in the context of individualised care.

## Background

### Prevalence of disability in women worldwide and in the UK

Based on the 2010 global population report more than one billion people have been estimated to have some form of disability, a total of 15% of the world’s population, with a prevalence of 10% estimated among women of childbearing age [1]. The proportion of individuals with disabilities is rising, possibly due to a changing age structure and to an increase in chronic health conditions [1]. Moreover, a higher prevalence of disability is reported among women, older people and low income families [2]. Many women of childbearing age have encountered significant difficulties in daily living because of a disabling condition [3]; and while disabled women have the same desire and legitimate right to become mothers as other women [4], their parenting ability is often brought into question [5]. More disabled women are having children [6]. A large scale UK population based study of women with a limiting longstanding illness, who had recently given birth found a prevalence of 9.4% of limiting longlasting illness [7].

### Defining disability – the WHO definition of disability

There is no universal agreement on a definition of disability. However, the International Classification of Functioning Disability and Health (ICF) defines disability as “an umbrella term, covering impairments, activity limitations, and participation restrictions”; adding that “disability is a contextual variable, dynamic over time and in relation to circumstances; its prevalence corresponds to social and economic status”. Disability is thus seen as “a complex phenomenon, reflecting an interaction between features of a person’s body and features of the society in which he or she lives” [1].

Increasing evidence suggests that disabled people in general have a poorer level of health, lower educational achievements and a higher rate of unemployment than their non-disabled counterparts [1]. Disability can be physical, mental, sensory, or involve a learning disability, it may be recent or long-term, progressive or stable and needs to be considered in terms of the physical implications and in relation to a woman’s coping abilities and those of her family [8].

Many disabled women have successfully become mothers and given birth to healthy babies [4]. However, barriers in access to health care providers and facilities have been reported for many women with physical disabilities [9]. High rates of abortion, miscarriage, caesarean section, and low usage of contraception were found in a survey involving 410 physically disabled women carried out in South Korea [10]. Increased risk of adverse pregnancy outcomes has been noted in women with some chronic illness, such as rheumatoid arthritis and schizophrenia [11]. For example, a higher proportion of low birth weight (LBW) babies (11.8% vs. 6.8%) has been reported for offspring of mothers with schizophrenia compared with other mothers [12].

To ensure a safe pregnancy and a healthy baby it is argued that healthcare professionals should focus more on women’s abilities than their disabilities [4] and that care and communication should be about empowering women. Evidence from qualitative research suggests that maternity care needs have not been met for many pregnant disabled women [5,13]. Many women with disabilities say they feel invisible in the healthcare system, stressing that their problems are not simply medical, but also social and political, and that access means more than mere physical accessibility [14]. Because many women with disabilities face a great deal of unpredictability in their daily lives, they want care that is well planned and which helps to eliminate the unexpected [15].

The recommendation of the current UK NICE Antenatal Care Guidelines is that all pregnant women should access health care services early. In general, people with disability may face considerable challenges in accessing health care services [16]. What little research exists on addressing maternity issues among disabled mothers generally focuses on their disability rather than their reproductive capability [17]. The aim, in this secondary analysis of population based survey data, was to obtain a picture of disabled women’s recent use and experience of maternity care and to better understand the issues arising with different types of disability.

## Methods

### Objectives

The specific objectives of this study were to:

• Describe the maternity care provided during pregnancy, birth and the postnatal period for women with a disability

• Explore disabled and non-disabled women’s perceptions of care received during pregnancy, birth and the postnatal period

• To compare the care and perceptions of the care received by women with different types of disability with those of women with no disclosed disability

### Study design

In England in 2010 data were collected for the Care Quality Commission (CQC) (which has a role in regulation, inspection and review of the operation of national standards in healthcare), by surveys in all 144 acute National Health Service Trusts (geographically based healthcare organisations) providing maternity services. A structured 12 page questionnaire, based on that used in previous national user surveys [18,19], with sections on pregnancy (31 questions), labour and birth (17 questions) and the postnatal period (21 questions). The postal survey was sent out to over 50,000 women aged 16 years (excluding women whose babies had died) three months after they had given birth. Completion and return of the questionnaire was taken as consent. The questionnaire covered access, information, communication and choice. Data on antenatal care, delivery mode, ratings of care and neonatal outcomes were collected as were socio-demographic characteristics including age, parity, ethnicity and partner status. Respondents’ ages were categorized as less than 20 years, 20–24, 25–29, 30–34 and 35 years or more. For ethnicity five categories were used: White, Mixed, Asian or Asian British, Black or Black British, Chinese or other ethnic group. The study, which complied with the Helsinki Declaration, involved secondary data analysis. The original survey evaluating maternity services in England was passed by the North West 5 Multi-Centre Research Ethics Committee (07/MRE8/1).

Respondents were asked if they had any long-standing conditions, with seven options including ‘No, I do not have a longstanding condition’. Five disability types were distinguished in the analyses: physical (long-standing physical condition and long-standing illness), sensory (deafness or severe hearing impairment and blindness or partial sightedness), mental, learning disability, and having a combination of two or more of these.

Women were asked questions about their antenatal care, labour and birth and postnatal care using three to five point Likert type scales. Women responded to a question on overall view of the three different phases of care rating on a five point scale: responses with the categories ‘excellent’, ‘very good’ and ‘good’ were combined and compared with those using the ‘ fair’ and ‘poor’ categories.

In the first part of this study we compared the maternity care received by women with disability and that received by women without disability. In the second part we examined the differences in maternity care received by the disabled women based on the type of disability.

### Statistical analyses

Descriptive statistics, including frequencies and proportions were calculated and Chi-squared tests of significance carried out. The Bonferroni method was applied to allow for multiple significance tests with adjusted p-values <0.01 regarded as statistically significant. Using logistic regression, adjustment for confounding variables, namely age, ethnicity, parity and partner status was undertaken. Analyses were performed separately for the disabled and non-disabled comparison and for the five disability subgroups described above. Women who did not complete the questions in the survey relating to disability were excluded. For all comparisons between a specific disability group and non-disabled women the statistical significance level was set at p = <0.01. The study sample was weighted for non-response based on the propensity modelling cell adjustment approach [20], and clustered by acute Trust (healthcare organisation). Robust standard errors were calculated using survey commands in Stata to account for the clusters at NHS trust level as well as non-response weights.

Statistical analyses were carried out using STATA 11 software (Stata Corp LP, College Station, TX).

## Results

### Socio-demographic characteristics

The response to the survey was 52%, representing 24,155 women in total. A comparison of the survey respondent characteristics with national data on women giving birth in England and Wales in 2010 [19] shows that they were more likely to be older (mean age 31 compared with 29.5 years), with 62% compared with 48% of mothers aged 30 years or more. However, there was little or no difference in parity or being partnered between the respondents and the wider population of women giving birth in England in the same year [21]. Slightly fewer women with low birthweight babies responded to the survey than was present in the 2010 population (5% compared with 7%) and the proportions of women having multiple births did not differ.

Disabled women comprised 6.1% (1,482) of the sample. A comparison of the age distribution of the disabled and non-disabled women shows that more disabled women were likely to be 35 years or older (32% vs. 29%) (Table 1), although the mean and median age for both groups was 31 years. There was no difference in parity, with nearly half of the women in both groups being primiparous (48% and 50%) or in being of White ethnicity (87% and 86%). Small differences were evident among the ethnicity groups due to the number of disabled women who came from Asian or Asian British ethnic groups (5% disabled vs. 7% non-disabled women). Disabled women were less likely to have a partner (79% vs. 87%) and a higher proportion delivered preterm (12.2% vs. 7.1%) or had a low birth weight (LBW) baby (8.3% vs. 4.8%).

**Table 1:** Characteristics of pregnant women with and without disability and their babies

**Characteristics**	**Women with disability**	**Women without disability**
	**(n = 1482)**	**(n = 22673)**
	**Frequency (%)**	**Frequency (%)**
Age group*		
<20	42 (2.8)	42 (2.8)
20–24	194 (13.1)	2745 (12.1)
25–29	358 (24.2)	5336 (23.5)
30–34	414 (28.00)	7594 (33.5)
35+	473 (31.9)	6526 (28.8)
**Parity**		
Primiparous	709 (48.0)	11215 (50.0)
Multiparous	764 (52.0)	11380 (50.0)
Ethnic group*		
White	1283 (87.0)	19375 (86.0)
Mixed	31 (2.0)	367 (2.0)
Asian or Asian British	73 (5.0)	1670 (7.0)
Black or Black British	70 (5.0)	880 (4.0)
Chinese or Other Ethnic Group	22 (1.0)	328 (1.0)
Partner**		
Yes	1164 (79)	19628 (87.0)
No	318 (21.0)	3045 (13.0)
Gestation at birth**		
> = 37 weeks	1286 (87.8)	20943 (92.9)
< 37 weeks	179 (12.2)	1593 (7.1)
**Birth weight** **		
> = 2500 g	1336 (91.7)	21347 (95.2)
<2500 g	121 (8.3)	1088 (4.8)
**Plurality**		
Single baby	1452 ( 98.4)	22211 (98.4)
Twins	22 (1.5)	353 (1.6)
Triplets or more	1 (0.1)	11 (0.1)

*p < 0.01, **p < 0.001.

### Access and care during antenatal, labour and birth and postnatal periods

Women with a disability were no different from non-disabled women in the timing of first contact with a health professional about their pregnancy care (94% and 95% by 12 weeks) or in their booking appointment (88% and 90% by 12 weeks) (Table 2). Disabled women however, had significantly more contact with antenatal services during the pregnancy: over a third had 10 or more antenatal checks compared with a fifth of non-disabled women (34% vs. 21%); half had four or more ultrasound scans in comparison with a third of non-disabled women (50% vs. 32%). Moreover, disabled women were significantly less likely to choose their place of birth (64% vs. 80%), and for more disabled women (17% vs. 5%) this was not possible for medical reasons. There was no difference in the proportion having a dating scan (95% in both groups); in being tested for Down’s syndrome (96% in both groups), or having a mid-trimester ultrasound scan, with 99% of all women being scanned to detect structural fetal anomalies. On the other hand, a significant difference was apparent in the proportion of women who attended antenatal classes (53% vs. 62%). This difference was not associated with preterm birth, and as with non-disabled women, being primiparous was associated with antenatal education attendance.

**Table 2:** Access and clinical care for disabled women during the antenatal, labour and birth and postnatal periods

	**Outcome**	**Women with disability n (%)**	**Women without disability n (%)**	**Adjusted odds ratio**
		**(n = 1482)**	**(n = 22673)**	**(95% CI)**
***Pregnancy***	First saw a health professional by 12 completed weeks	1350 (94.3)	21115 (95.3)	1.12 (0.87- 1.44)
	Booking appointment by 12 completed weeks	1153 (88.2)	18484 (89.5)	1.09 (0.91-1.31)
	10 or more antenatal checks **	455 (34.2)	4376 (21.1)	**1.88 (1.66-2.12)**
	4 or more antenatal scans during pregnancy**	729 (50.2)	7223 (32.2)	**2.04 (1.83-2.28)**
	Dating scan at 8–14 weeks of pregnancy	1378 (95.3)	21253 (95.2)	0.93 (0.71-1.21)
	Testing for down’s syndrome	1032 (96.4)	16119 (96.8)	1.06 (0.74-1.53)
	Mid-trimester (anomaly/20 weeks) scan	1439 (99.2)	22143 (99.0)	0.82 (0.45-1.50)
	Attended NHS antenatal classes (of those wishing to)**	463 (53.0)	8193 (62.4)	**0.71 (0.58-0.86)**
***Labour and birth***	Had met staff before labour**	430 (30.0)	5191 (23.3)	**0.72 (0.63-0.81)**
	Had choice of place of birth **	899 (64.4)	17329 (80.1)	**1.44 (1.23-1.67)**
	Given epidural for pain relief in labour	379 (31.5)	5746 (28.9)	**0.85 (0.74-0.97)**
	Given pethidine for pain relief in labour	371 (30.8)	5948 (30.0)	0.96 (0.84-1.10)
	Able to choose a comfortable position most of the time **	579 (48.6)	11308 (57.3)	0.72 (0.63-0.81)
	Caesarean section birth**	445 (30.5)	5437 (24.2)	**1.35 (1.19-1.52)**
***Postnatal care***	Length of postnatal stay > =3 days**	564 (39.6)	6230 (28.6)	**1.63 (1.45-1.83)**
	Baby put to breast at least once**	1131 (77.3)	18987 (84.4)	**1.41 (1.23-1.66)**
	Full or partial breast milk feeds in first few days **	1018 (70.1)	17814 (79.4)	**0.65 (0.57-0.74)**
	Total number of postnatal midwife contacts > = 5*	383 (27.0)	5260 (24.0)	**1.22 (1.08-1.39)**
	Postnatal check	1279 (87.5)	19927 (89.0)	1.11 (0.93-1.31)
	Given advice about contraception	1306 (90.3)	20569 (92.2)	**0.78 (0.65-0.95)**

*p < 0.01, **p < 0.001 Adjustment for age, parity, ethnicity and partner status Significant OR in bold.

With regard to labour and birth a significantly higher proportion of disabled women (30% vs. 23%) had met the staff caring for them before labour. In relation to pain relief there was no difference in disabled and non-disabled women’s use of pethidine (or similar medication) (31% vs. 30%) or in use of epidural anaesthesia (or similar) (32% vs. 29%). Disabled women were significantly more likely to have a caesarean section (31% vs. 24%) and this was likely to have been a planned procedure (48% vs. 41%, p = <0.001) (data not shown). Significantly fewer women with disabilities were able to choose a comfortable position most of the time during labour compared with non-disabled women (49% vs. 57%) and for more disabled women this was not possible at all (15% vs. 12%) (data not shown).

Postnatally, disabled women were significantly more likely to stay in hospital for three or more days (40% vs. 29%), were significantly less likely to put their baby to the breast at least once (77% vs. 84%) and to breastfeed partially or exclusively during the first few days (70% vs. 79%). Once home, disabled women were more likely to have seen a midwife five or more times (27% vs. 24%). Around 90% of women in both groups received postnatal checks and contraceptive advice.

### Perceptions of maternity care

In assessing quality of care, women were asked about information, staff attitudes and communication during the different phases of their care (Table 3).

**Table 3:** Perceptions of care for disabled and non-disabled women

	**Outcome**	**Women with disability n (%)**	**Women without disability n (%)**	**Adjusted odds ratio**
		**(n = 1482)**	**(n = 22673)**	**(95%CI)**
***Pregnancy***	Always spoken to in a way you could understand**	1156 (79.0)	18807 (83.8)	**0.74 (0.65-0.85)**
	Always involved enough in decisions about your care**	993 (68.1)	16469 (73.9)	**0.76 (0.67-0.85)**
	If contacted midwife, always given help when needed**	784 (67.4)	11775 (72.8)	**0.77 (0.67-0.89)**
	Antenatal care overall rating ‘Good’ (excellent/very good/good)	1344 (91.8)	20830 (92.6)	0.93 (0.76-1.14)
***Labour and birth***	Partner/companion definitely welcomed**	1157 (80.9)	18677 (84.4)	**0.8 (0.70-0.92)**
	Definitely had confidence and trust in staff providing care**	1016 (69.7)	16617 (74.0)	**1.2 (1.06-1.35)**
	Always spoken to in a way you could always understand**	1168 (80.0)	18914 (84.3)	**1.34 (1.16-1.54)**
	Always involved enough in decisions about care**	936 (65.3)	15917 (70.6)	**0.74 (0.66-0.84)**
	Definitely received the pain relief you wanted *	641 (56.3)	11356 (60.5)	**0.84 (0.74-0.95)**
	Not left alone and worried during labour**	1165 (80.5)	18824 (84.4)	**1.31 (1.14-1.51)**
	Labour and birth care overall rating ‘Good’ **	1327 (91.1)	20971 (93.6)	**1.35 (1.11-1.64)**
***Postnatal care***	Postnatal care in hospital always treated with kindness and understanding*	845 (59.5)	13869 (63.6)	**1.18 (1.05-1.33)**
	Postnatal care in hospital always given information/explanations needed	706 (49.6)	11555 (52.9)	**1.14 (1.02-1.28)**
	Felt length of stay about right	962 (68.8)	15497 (72.6)	**0.83 (0.73-0.930**
	At home if contacted midwife/health visitor, help always given**	730 (70.4)	11466 (75.6)	**0.77 (0.67-0.89)**
	Would have liked more midwife visits**	356 (25.1)	4624 (20.9)	**0.78 (0.68-0.89)**
	Definitely given enough information about recovery after birth	836 (58.5)	12680 (58.1)	0.97 (0.86-1.08)
	Definitely given enough information about emotional changes	591 (42.6)	8876 (42.2)	1.03 (0.92-1.16)
	Always had active support and encouragement with feeding	620 (43.8)	9529 (43.8)	1.01 (0.90-1.14)
	Always had consistent advice about infant feeding	511 (36.5)	7777 (36.1)	1.01 (0.90-1.14)
	Postnatal care overall rating ‘Good’*	1254 (85.8)	19858 (88.5)	**1.23 (1.05-1.45)**

*p < 0.01, **p < 0.001 Adjustment for age, parity, ethnicity and partner status Significant OR in bold.

During pregnancy, significantly fewer pregnant disabled women reported always being spoken to in a way they could always understand (79% vs. 84%) and always being involved in decisions about their antenatal care (68% vs. 74%) (Table 3). Moreover, a significant proportion of disabled women reported that when they had contacted a midwife they were not always given the help they required (33% vs. 27%). However, almost all women in both groups (92% and 93%) rated their care as ‘good’ or better than ‘good’.

During labour, disabled women were less likely to say that their companion or partner was definitely welcomed (81% vs. 84%). They were also less likely to report definitely having confidence and trust in the staff caring for them at this time (70% vs. 74%) and always being spoken to in a way they could understand (81% vs. 84%). Fewer women with disabilities reported always being involved in decisions about their care (65% vs. 71%) and those who required pain relief during labour and birth were less likely to report receiving the pain relief they wanted (56% vs. 61%). Slightly more felt that at some time they had been left alone and worried during labour and birth (19% vs. 16%) and overall were slightly less likely to rate their care at this time as ‘good’ or better compared with non-disabled women (91% vs. 94%).

Following birth, while still in hospital, mothers with disability were less likely to report always being treated with kindness and understanding (60% vs. 64%). However, there were no differences in relation to perceptions of length of stay or being given information or explanations about their physical recovery or emotional changes associated with birth. For care received at home, fewer disabled women reported that contacting a midwife was always helpful (70% vs. 76%) and more would like to have seen a midwife more often (25% vs. 21%). Regarding infant feeding, in both groups active support from health professionals was always received by less of half of women (44%) and consistent advice by just over a third (36%). When disabled women were asked to rate their care after birth, slightly fewer disabled women rated their postnatal care as good or better (86% vs. 89%).

The significant differences reported for this disabled/non-disabled comparison in care and perceptions of care, were maintained in the adjusted analysis. Additionally, in the adjusted analysis significantly more women with disabilities reported having an epidural for pain relief in labour (OR = 0.85, 95% CI 0.74-0.97).

### Differences between different types of disabilities

In this analysis five disability types were distinguished: of those 1482 women who self-identified as disabled almost half had a physical disability (including chronic diseases and long-standing conditions (49%); nearly a quarter had a mental health condition (23%); and smaller proportions had a sensory impairment (being deaf, blind or partially sighted) (13%), a learning disability (8%) or more than one of the previous types of disability (7%).

### Women with physical disability

Women with a physical disability consisted of women with a long-standing health problem involving a physical condition, for example cerebral palsy, or a longstanding illness such as asthma or epilepsy (Table 4). The proportion of women with physical disability aged 35 years or more was twice that of non-disabled women (44% vs. 23%). Most of the disabled women were white (84%) and they were slightly less likely to have a partner (83% vs. 87%). Mothers with physical disability were significantly more likely to deliver their babies preterm (12.7% vs. 7.1%) and to have a LBW baby (7.9% vs. 4.8%).

**Table 4:** Characteristics of women with different types of disability and their babies compared with non-disabled women and their babies

	**Disability**					** No disability**
	**Physical condition or illness**	**Sensory**	**Mental health disability**	**Learning disability**	**More than one disability**	
**Characteristics**	n = 730 n (%)	n = 197 n (%)	n = 336 n (%)	n = 120 n (%)	n = 99 n (%)	(n = 22,673) n (%)
**Age group** (years)	**			**		
<20	12 (1.6)	9 (4.6)	9 (2.7)	8 (6.8)	4 (4.04)	470 (2.1)
20–34	394 (54.0)	123 (62.4)	220 (65.5)	93 (77.5)	64 (64.7)	16990 (74.9)
35+	324 (44.4)	65 (33.0)	107 (31.9)	19 (15.8)	31 (31.3)	5213 (23.0)
**Ethnic group**	**		*			
White	610 (83.6)	172 (87.8)	313 (93.4)	106 (88.3)	82 (83.7)	19375 (85.7)
Mixed	15 (2.1)	5 (2.6)	6 (1.8)	3 (2.5)	2 (2)	367 (1.6)
Asian or Asian British	41 (5.6)	12 (6.1)	9 (2.7)	3 (2.5)	8 (8.2)	1670 (7.4)
Black or Black British	52 (7.1)	5 (2.6)	6 (1.8)	4 (3.3)	3 (3.1)	880 (3.9)
Chinese or Other	12 (1.6)	2 (1.0)	1 (0.3)	4 (3.3)	3 (3.1)	328 (1.5)
**Parity**						
Primiparous	351 (48.3)	101 (51.5)	145 (43.2)	67 (57.8)	45 (45.5)	11215 (49.6)
Multiparous	375 (51.7)	95 (48.5)	191 (56.8)	49 (42.2)	54 (54.5)	11380 (50.4)
**Partner**	*	*	**	**	**	
Yes	607 (83.2)	157 (79.7)	253 (75.3)	82 (68.3)	65 (65.7)	19628 (86.6)
No	123 (16.8)	40 (20.3)	83 (24.7)	38 (31.7)	34 (34.3)	3045 (13.4)
**Language at home**			**			
English	647 (90.9)	158 (83.2)	323 (96.7)	104 (90.4)	90 (90.9)	19789 (88.4)
Other	65 (9.1)	32 (16.8)	11 (3.3)	11 (9.6)	9 (9.1)	2588 (11.6)
**Gestation at birth**	**	*	*		**	
> = 37 weeks	630 (87.3)	172 (87.8)	298 (89.2)	107 (92.2)	79 (81.4)	20943 (92.9)
< 37 weeks	92 (12.7)	24 (12.2)	36 (10.8)	9 (7.8)	18 (18.6)	1593 (7.1)
**Birth weight**	**		*		**	
> = 2500 g	664 (92.1)	177 (93.2)	303 (91.3)	108 (93.1)	84 (85.7)	21347 (95.2)
<2500 g	57 (7.9)	13 (6.8)	29 (8.7)	8 (6.9)	14 (14.3)	1088 (4.8)

*p < 0.01, **p < 0.001 Sensory disability: visually impaired, deaf and hearing impaired.

In terms of accessing services and clinical care during pregnancy, like the non-disabled group, around 95% women with a physical disability contacted a health care professional about their pregnancy at or before week 12 (Table 5). Similarly, three quarters of both groups had also made their booking appointment by this point in time. However, women with a long term physical illness or conditions were almost twice as likely to have 10 or more antenatal checks (41% vs. 21%) and 4 or more ultrasound scans during pregnancy (59% vs. 32%), although they were no more likely to have the standard antenatal screening. A lower and significant proportion of women with disability attended antenatal classes (53% vs. 62%), and this was unrelated to prematurity (p = 0.27) (data not shown).

**Table 5:** Antenatal care received and perceptions of care for women with different types of disability

	**Physical condition & illness**	**Sensory**	**Mental health disability**	**Learning disability**	**More than one disability**	**No disability**
***Antenatal care received***	(n = 730) n (%)	(n = 197) n (%)	(n = 336) n (%)	(n = 120) n (%)	(n = 99) n (%)	(n = 22,673) n (%)
First saw health professional by 12 weeks	682 (96.3)	175 (93.1)	311 (94.8)	94 (84.7) **	88 (90.7)	21115 (95.3)
aOR (95% CI)	0.75 (0.50-1.14)	1.51 (0.83- 2.74)	0.85 (0.49-1.47)	**3.29 (1.88-5.77)**	1.38 (0.64-2.97)	1
Booking appointment by 12 weeks	495 (76.7)	129 (73.7)	226 (73.9)	64 (67.4)	63 (72.4)	15633 (75.7)
aOR (95% CI)	0.93 (0.77-1.13)	1.1 (0.77-1.57)	1.08 (0.83-1.41)	1.51 (0.96-2.38)	1.19 (0.73-1.94)	1
10 or more antenatal checks	274 (40.7)**	44 (25.1)	85 (28.7)*	20 (20.2)	32 (36.8)**	4376 (21.1)
aOR (95% CI)	**2.51 (2.13-2.96)**	1.26 (0.88-1.81)	**1.48 (1.14-1.93)**	0.8 (0.46-1.39)	**1.98 (1.24-3.17)**	1
4 or more scans during pregnancy	419 (58.5) **	68 (36.4)	156 (46.6) **	38 (32.5)	48 (49.5) **	7223 (32.2)
aOR (95% CI)	**2.83 (2.42-3.30)**	1.18 (0.86-1.60)	**1.83 (1.46-2.29)**	0.96 (0.64-1.46)	**1.89 (1.25-2.85)**	1
Dating scan at 8–14 weeks of pregnancy	690 (96.2)	180 (93.8)	315 (95.2)	102(90.3)	91(97.8)	21253 (95.2)
aOR (95% CI)	0.69 (0.46-1.06)	1.63 (0.90-2.96)	0.93 (0.55-1.60)	1.56 (0.78-3.11)	0.47 (0.11-2.04)	1
Testing for Down’s syndrome	534 (96.9)	133 (95.7)	226 (96.2)	73 (93.6)	66 (97.1)	16119 (96.8)
aOR (95% CI)	1.06 (0.61-1.82)	1.19 (0.48-2.98)	0.97 (0.48-1.95)	1.42 (0.54-3.78)	0.75 (0.18-3.14)	1
Mid-trimester (anomaly/20 weeks) scan	717 (99.4)	185 (98.9)	329 (98.8)	114 (100)	94 (97.9)	22143 (99)
aOR (95% CI)	0.52 (0.19-1.41)	1.33 (0.33-5.35)	1.43 (0.51-4.04)	NA	1.22 (0.27-5.54)	1
Attended NHS antenatal classes	230 (52.6) **	68 (61.3)	103 (52.3) *	39 (56.5)	23 (39) **	8193 (62.4)
aOR (95% CI)	**1.54 (1.16-2.0)**	0.77 (0.43- 1.41)	1.16(0.78-1.72)	1.77 (0.94- 3.31)	**3.2 (1.68-6.17)**	1
***Maternal perceptions of antenatal care***						
Always spoken to in a way you could understand	604 (83.4)	149 (78.8)	258 (77.2)*	78 (65.5)**	67 (69.1)**	18807 (83.8)
aOR (95% CI)	0.97 (0.78-1.19)	0.72 (0.50-1.05)	**0.67 (0.51-0.87)**	**0.42 (0.28-0.64)**	**0.51 (0.32-0.83)**	1
Always involved enough in decisions about care	506 (70.3)	136 (73.1)	227 (68.6)	73 (62.4)**	51 (53.1)**	16469 (74.4)
aOR (95% CI)	**0.83 (0.70-0.98)**	0.88 (0.63-1.23)	**0.74 (0.58-0.94)**	0.67 (0.45-1.01)	**0.42 (0.27-0.64)**	1
If contacted midwife, always given help when needed	392 (70.0)	99 (70.2)	189 (65.6) *	55 (57.9)*	49 (61.3)	11775 (72.8)
aOR (95% CI)	0.85 (0.71-1.03)	0.93 (0.63-1.35)	**0.69 (0.53-0.88)**	**0.63 (0.40-0.99)**	0.66 (0.40-1.09)	1
Antenatal care overall rating ‘Good ‘	669 (92.7)	182 (93.8)	294 (88.6) *	111 (93.3)	88 (90.7)	20830 (92.6)
aOR (95% CI)	1.0 (0.74-1.35)	1.06 (0.57-2.00)	**0.65 (0.45-0.93)**	1.55 (0.70-3.40)	1.01 (0.49-2.07)	1

*p < 0.01, **p < 0.001 Sensory disability: visually impaired, deaf and hearing impaired Significant OR in bold.

Perceived quality of care during pregnancy did not differ significantly for this group of women with a physical condition or health problem: over 80% of respondents in both groups felt they were talked in a way they could understand; 70% or more felt they had been involved enough in decisions about their care given and help when needed. More than 90% rated their antenatal care as ‘good’ or better (Table 5).

Similar results concerning antenatal care were still evident after adjustment. However, a further significant difference was found with more physically disabled women reporting insufficient involvement in their care at this time (OR = 0.83, 95% CI 0.70-0.98).

With regard to labour most women had not met the staff caring for them before, however, women with a physical disability were more likely to have done so (30% vs. 23%) (Table 6). They were significantly less likely to report having a choice of place of birth (59% vs. 80%), although for some this was for medical reasons (24% vs. 5%) (p = <0.001) (data not shown). Fewer women with a physical disability or long term condition reported being able to choose a comfortable position in labour (43% vs. 57%), with some not being able to do so at all (19% vs. 12%). For pain relief in labour this group were significantly more likely to have epidural anaesthesia (36% vs. 30%) and to give birth by caesarean section (38% vs. 24%). Overall perceptions of labour and birth care were largely positive, with very few differences, the exception being that women with a physical disability or long term condition were significantly less likely to report feeling involved enough in decision-making about their care (65% vs. 72%).

**Table 6:** Labour and birth care received and perceptions of care for women with different types of disability

	**Disability**					**No disability**
	**Physical condition & illness**	**Sensory**	**Mental health disability**	**Learning disability**	**More than one disability**	
***Labour and birth care received***	(n = 730) n (%)	(n = 197) n (%)	(n = 336) n (%)	(n = 120) n (%)	(n = 99) n (%)	(n = 22,673) n (%)
Had met staff before labour	213 (30.0) **	61 (33.3) *	76 (23.3)	39 (33.6) *	41 (42.3) **	5191 (23.3)
aOR (95% CI)	**0.74 (0.62-0.88)**	**0.58 (0.42-0.80)**	0.95 (0.73-1.25)	**0.6 (0.39-0.90)**	**0.48 (0.31-0.75)**	1
Had choice of place of birth	406 (58.5) **	136 (74.7)	220 (69.6) **	84 (73.0)	53 (58.9) **	17329 (80.1)
aOR (95% CI)	**1.48 (1.19- 1.84)**	1.14 (0.75- 1.72)	**1.53 (1.14- 2.05)**	1.26 (0.76-2.07)	1.76 (1.00- 3.08)	1
Able to choose a comfortable position in labour	229 (42.7)**	93 (55.7)	161 (54.8)	59 (52.2)	37 (45.1)	11308 (57.2)
aOR (95% CI)	**1.82 (1.52-2.81)**	0.96 (0.69-1.33)	1.12 (0.88-1.43)	1.12 (0.75-1.67)	1.55 (0.97-2.47)	1
Given epidural for pain relief in labour	193 (35.5) *	41 (24.6)	93 (31.1)	30 (26.5)	22 (26.8)	5746 (28.9)
aOR (95% CI)	**0.71 (0.59-0.86)**	1.25 (0.84-1.86)	0.81 (0.62-1.05)	1.03 (0.65-1.63)	1.15 (0.67-1.97)	1
Given pethidine for pain relief in labour	159 (29.2)	55 (32.9)	104 (34.8)	30 (26.5)	23 (28.0)	5948 (29.9)
aOR (95% CI)	1.01 (0.83-1.24)	0.89 (0.63-1.25)	**0.76 (0.59-0.97)**	1.4 (0.88-2.25)	1.15 (0.67-1.96)	1
Caesarean section birth	271 (37.6) **	47 (24.4)	77 (23)	21 (18.1)	29 (30.2)	5437 (24.2)
aOR (95% CI)	**1.84 (1.52-2.24)**	0.74 (0.49-1.13)	1.07 (0.81-1.44)	0.90 (0.56-1.46)	1.38 (0.82-2.33)	1
***Maternal perceptions of labour and birth care***						
Partner/companion definitely welcomed	577 (81.4)	159 (84.6)	257 (79.3)	92 (78.6)	72 (77.4)	18677 (84.4)
aOR (95% CI)	1.06 (0.62-1.80)	**2.19 (1.07-4.46)**	1.6 (0.87-2.95)	1.4 (0.50-3.95)	1.8 (0.69-4.70)	1
Definitely had confidence and trust in staff	526 (72.8)	134 (70.2)	225 (68.4)	72 (61.5)*	59 (60.2)*	16617 (74.0)
aOR (95% CI)	1.36 (0.95-1.94)	1.37 (0.73-2.57)	**1.88 (1.18-3.00)**	1.75 (0.86-3.57)	**3.05 (1.56-5.95)**	1
Always spoken to in a way you could understand	592 (82.5)	157 (81.3)	268 (80.5)	82 (68.9)**	69 (71.1)**	18914 (84.3)
aOR (95% CI)	1.06 (0.69-1.63)	0.96 (0.68-1.36)	**0.59 (0.37-0.95)**	0.66 (0.39-1.13)	1.23 (1.00-1.50)	1
Always involved enough in decisions about care	458 (64.7)**	134 (71.7)	217 (67.0)	72 (61.0)*	55 (56.7)*	15917 (72.1)
aOR (95% CI)	0.78 (0.57-1.06)	0.97 (0.54-1.73)	**0.57 (0.39-0.85)**	**1.05 (0.52-2.13)**	**0.37 (0.21-0.66)**	1
Definitely received the pain relief you wanted	287 (55.8)	94 (60.3)	163 (56.2)	59 (56.7)	38 (50.7)	11356 (60.5)
aOR (95% CI)	1.27 (1.00-1.60)	0.85 (0.54-1.34)	**1.62 (1.21-2.15)**	0.68 (0.37-1.25)	**2.09 (1.24-3.53)**	1
Not left alone and worried during labour	599 (83.4)	150 (78.1)	258 (79.1)	87 (75.0)*	71 (74.7)	18824 (84.4)
aOR (95% CI)	1.1 (0.90-1.36)	1.51 (1.06-2.17)	1.47 (1.11-1.94)	**1.62 (1.04-2.53)**	1.59 (0.94-2.67)	1
Labour and birth care overall rating ‘Good’	662 (92.1)	182 (93.8)	296 (90.8)	108 (90.8)	79 (79.8)**	20971 (93.6)
aOR (95% CI)	0.79 (0.59-1.05)	1.25 (0.68-2.30)	**0.66 (0.44-0.98)**	0.89 (0.46-1.73)	**0.35 (0.20-0.61)**	1

*p < 0.01, **p < 0.001 Sensory disability: visually impaired, deaf and hearing impaired Significant OR in bold.

Postnatal care for physically disabled women involved significantly longer postnatal stays (45% vs. 29% staying for 3 days or more) (Table 7). There were also some differences in early infant feeding: physically disabled were women significantly less likely to put the baby to the breast at least once (80% vs. 84%) and to go on to breastfeed in the first few days (74% vs. 79%). Once home, there were no differences in midwife contacts, receiving a postnatal check or advice about contraception.

**Table 7:** Postnatal care received and perceptions of care for women with different types of disability

	**Disability**					**No disability**
	**Physical condition & Illness**	**Sensory**	**Mental disability**	**Learning disability**	**More than one disability**	
***Postnatal care received***	(n = 730)	(n = 197)	(n = 336)	(n = 120)	(n = 99)	(n = 22,673)
	n (%)	n (%)	n (%)	n (%)	n (%)	n (%)
Length of postnatal hospital stay < 3 days	316 (44.7) **	59 (31.2)	110 (34.2)	34 (30.6)	45 (46.9) **	6230 (28.6)
aOR (95% CI)	**1.92 (1.64-2.25)**	1.13 (0.81 -1.58)	**1.43 (1.12-1.82)**	1.14 (0.75-1.74)	**2.18 (1.42-3.35)**	1
Baby put to breast at least once	576 (79.8)*	153 (78.9)	245 (73.6)**	86 (74.1)*	71 (72.5)*	18987 (84.4)
aOR (95% CI)	**1.47 (1.18-1.83)**	1.28 (0.87 -1.86)	**1.48 (1.13-1.95)**	1.29 (0.81 -2.03)	1.51 (0.86 -2.65)	1
Full or partial breast milk feeds in first few days	529 (73.5 )**	133 (69.2)*	223 (67.3)**	75 (65.2)**	58 (60.4)**	22001 (79.4)
aOR (95% CI)	0.94(0.64-1.39)	0.98 (0.72-135)	0.98 (0.63-1.53)	0.71 (0.43-1.15)	**1.48 (1.22-1.79)**	1
Total number of postnatal midwife contacts > = 5	166 (24.0)	61 (33.0) *	98 (31.0)*	35 (31.3)	23 (25.8)	5260 (24.0)
aOR (95% CI)	1 (0.83-1.20)	**1.48 (1.08-2.05)**	**1.49 (1.16-1.91)**	**1.6 (1.03-2.47)**	1.16 (0.70-1.94)	1
Received postnatal check	638 (88.5)	173 (89.2)	283 (85.5)	99 (83.9)	86 (87.8)	19927 (89.0)
aOR (95% CI)	1.07 (0.84-1.36)	0.89 (0.55-1.44)	1.32 (0.96-1.80)	1.27 (0.74-2.16)	0.94 (0.49-1.80)	1
Given advice about contraception	640 (89.6)	176 (92.2)	296 (89.4)	110 (95.7)	84 (88.4)	20569 (92.2)
aOR (95% CI)	1.35 (0.73-2.50)	1.0 (0.64-1.55)	**3.01 (1.16-7.81)**	0.8 (0.39-1.64)	**1.39 (1.07-1.79)**	1
***Perceptions of postnatal care***						
Always treated with kindness and understanding in hospital	422 (60.0)	116 (62.4)	192 (59.3)	64 (57.7)	51 (53.7)	13869 (63.6)
aOR (95% CI)	0.85 (0.72-1.00)	1 (0.73-1.38)	0.79 (0.63-1.00)	0.89 (0.59-1.34)	0.7 (0.46-1.08)	1
Always given information/explanations needed in hospital	345 (49.0)	105 (56.1)	159 (48.6)	54 (48.2)	43 (45.7)	11555 (52.9)
aOR (95% CI)	**0.85 (0.72-0.99)**	1.18 (0.87-1.60)	**0.78 (0.62-0.98)**	0.95 (0.63-1.42)	0.85 (0.55-1.31)	1
Felt length of stay about right	494 (71.3)	128 (69.6)	219 (68.4)	63 (57.8)*	58 (63.0)	15497 (72.6)
aOR (95% CI)	0.93 (0.78-1.10)	0.85 (0.61-1.19)	0.82 (0.64-1.05)	**0.51 (0.34-0.77)**	0.68 (0.43-1.06)	1
At home if contacted midwife/health visitor, help always given	356 (71.3)	98 (71.5)	167 (70.5)	61 (69.3)	48 (63.2)	11466 (75.6)
aOR (95% CI)	0.88 (0.56-1.38)	0.55 (0.26-1.14)	0.93 (0.48-1.80)	3.74(0.51-27.2)	**0.41 (0.19-0.88)**	1
Would have liked to see a midwife more	180 (25.7)*	49 (26.1)*	66 (20.2)	33 (28.7)*	28 (31.1)*	4624 (20.9)
aOR (95% CI)	**0.78 (0.65-0.94)**	**0.67 (0.48-0.93)**	0.96 (0.72-1.28)	0.72 (0.47-1.11)	0.63 (0.39-1.04)	1
Definitely given enough information about recovery after birth	292 (41.1)	92 (49.5)	118 (36.9)	51 (44.3)	40 (41.2)	9154 (41.9)
aOR (95% CI)	0.83 (0.69-1.02)	1.21 (0.80-1.85)	**0.6 (0.46-0.77)**	1.04 (0.63-1.70)	**0.54 (0.33-0.88)**	1
Definitely given enough information about emotional changes	280 (41.1)	92 (49.7)	132 (41.4)	49 (44.1)	38 (42.2)	8876 (42.2)
aOR (95% CI)	0.85 (0.70-1.02)	0.96 (0.66-1.38)	0.95 (0.72-1.25)	0.86 (0.53-1.37)	**0.52 (0.32-0.83)**	1
Always had active support and encouragement with feeding	301 (42.6)	98 (52.7)	133 (42.0)	46 (40.7)	42 (44.2)	9529 (43.8)
aOR (95% CI)	**0.80 (0.65-0.98)**	1.58 (0.92-2.71)	0.81 (0.60-1.10)	1.08 (0.63-1.85)	0.63 (0.37-1.06)	1
Always had consistent advice about infant feeding	246 (35.4)	86 (46.7)*	105 (33.0)	41 (36.9)	33 (35.1)	7777 (36.1)
aOR (95% CI)	**0.78 (0.61-0.98)**	**0.59 (0.39-0.88)**	**0.69 (0.50-0.93)**	0.82 (0.47-1.42)	**0.56 (0.32-0.98)**	1
Postnatal care overall rating ‘Good’	611 (85.0)*	177 (91.2)	284 (85.8)	106 (88.3)	76 (77.6)*	19858 (88.5)
aOR (95% CI)	**0.75 (0.60-0.93)**	1.27 (0.74-2.15)	0.78 (0.57-1.07)	1.18 (0.64-2.16)	**0.54 (0.33-0.90)**	1

*p < 0.01, **p < 0.001 Sensory disability: visually impaired, deaf and hearing impaired Significant OR in bold.

In terms of perceptions of postnatal care, about 70% in both groups felt the length of their hospital stay was about right. Smaller, but similar proportions of both groups reported always being treated with kindness and understanding (Table 7). Following adjustment, more physically disabled women reported not having been given the information needed at this time (OR = 0.85, 95% CI 0.72-0.99).

Once home, over 70% of women in both groups reported being given the help needed although, a significant number of women with a physical disability or long term condition would have liked to have seen a midwife more often (26% vs. 21%). There were no differences in relation to information about recovery and emotional changes following childbirth or advice about infant feeding. However, adjusted analysis showed significantly more women with physical disability did not always receive active support and encouragement with feeding compared with non-disabled women (OR = 0.8, 95% CI 0.65-0.98). Overall, postnatal care was significantly less likely to be rated as good or better among women with a physical disability (85% vs. 89%).

### Women with sensory impairment

The experiences of 197 women with a sensory disability (visual or hearing impairment) were compared with those of non-disabled women. Maternal characteristics such as age, ethnicity, language spoken at home and parity did not differ significantly (Table 4). However, women in this group were significantly less likely to have a partner (80% vs. 87%) and more likely to deliver preterm (12% vs. 7%).

For this group of women there were no differences in the antenatal care received or in the perceptions of that care, including aspects of communication and decision-making, with 94% of women with sensory impairment rating antenatal care as good (Table 5). Similarly for labour and birth there were practically no differences between the group with sensory impairment and the non-disabled women in the care received and perceptions of care, with the exception that women with a sensory impairment were significantly more likely to have previously met the staff caring for them (33% vs. 23%) (Table 6). Like the non-disabled women, almost all of those with sensory disabilities rated labour and birth care positively. Postnatally, this group of women were significantly more likely to have received advice about infant feeding (47% vs. 36%) but were less likely to breast-feed (69% vs. 79%). They were also significantly more likely to have seen the midwife at least five times (33% vs. 24%), but would like to have seen them more (26% vs. 21%, p < 0.01). Just over 90% of this group rated postnatal care as good or better. Similar findings were observed after adjustment.

### Women with mental health disability

For this group of 336 women self-identifying with mental health problems there was a significant difference in ethnicity in comparison with the non-disabled women, with fewer women in this group coming from Asian or Asian British and Black or Black British groups and more speaking English at home (Table 4). They were significantly less likely to have a partner (75% vs. 87%) and were at significantly higher risk of delivering a preterm (11% vs. 7%) or LBW baby (9% vs. 5%).

Antenatally almost all the women in both groups had seen a health professional about their pregnancy care by 12 weeks (Table 5) and approximately three quarters (74%) had attended their booking appointment by 12 weeks. Significantly greater numbers of women with mental health conditions had 10 or more antenatal checks (29% vs. 21%) and had 4 or more ultrasound scans (47% vs. 32%) although significantly fewer attended antenatal classes (52% vs. 62%). This group was significantly less likely to feel talked to in a way they could understand (77% vs. 84%), that the midwifery help needed was less likely to be provided (66% vs. 73%) and the proportion rating antenatal care as good was lower than that of the non-disabled women (89% vs. 93%).

Significantly fewer women with mental health problems reported having a choice about the location of birth (70% vs. 80%), but otherwise there were no differences in the care provided during labour and birth including type of delivery and pain relief given or in the women’s perceptions of care at this time compared with non-disabled women (Table 6). However, following adjusted analysis a significant difference was found with more women in this group rating their labour and birth care as poor (OR = 0.44, 95% CI 0.44-0.98).

Few significant differences with regard to postnatal care were found, however, mothers with mental health problems were more likely to have 5 or more midwife contacts (31% vs. 24%) and were significantly less likely to put the baby to the breast at all (74% vs. 84%) or to breastfeed in the first few days (67% vs. 79%) (Table 7). Additionally, following adjusted analysis women with mental disability were significantly less likely to report that they always had been given information and explanation needed in hospital (OR = 0.78, 95% CI 0.62-0.98).

### Women with learning disability

For this group of 120 women with learning disability the proportion with partners at the time of the survey was significantly less than for the non-disabled women (68% vs. 87%). Like the non-disabled women, most were aged between 20 and 34 years, however this group was slightly more likely to be represented among the youngest women aged less than 20 years of age (7% vs. 2%) (Table 4). No difference was found by parity or in the proportion giving birth to preterm or LBW babies.

Women with learning disability were significantly less likely to see a health professional by 12 weeks gestation (85% vs. 95%) and were no more likely to have higher numbers of antenatal checks, scans or screening than non-disabled women (Table 5). However, perceptions of care were less positive, with markedly fewer learning disabled women feeling that they were always spoken to in a way they could understand (66% vs. 84%), involved in decisions about their care (63% vs. 74%) and always given help after contacting a midwife (58% vs. 73%) compared with non-disabled women. Although this group were less likely to have positive views of specific aspects of their pregnancy care, almost all rated their overall antenatal care as good or better (93%).

For labour and birth, significantly more learning disabled women had met the staff caring for them previously (34% vs. 23%) although proportions were quite low for most groups (Table 6). No significant differences were found in the use of pain relief or in mode of delivery. Learning disabled women were more likely to be critical about specific aspects of their care: significantly fewer definitely had confidence and trust in staff (62% vs. 74%) and fewer were always spoken to in a way they could understand (68% vs. 84%). However, significantly fewer were not left alone and worried during labour (75% vs. 84%) and there was no difference in the overall rating of care.

With regard to postnatal care, this group was no more likely to have stayed 3 days or more than the non-disabled group and significantly fewer agreed that their length of stay was about right (58% vs. 73%). Initiation and continued breastfeeding in the first few days were significantly less common among the learning disabled women, a significantly greater proportion of whom would liked to have seen a midwife more (29% vs. 21%). Communication and information during this phase of care, as well as the overall rating, were no different between the two groups. Following adjustment similar findings on all the data items listed were observed.

### Women with more than one type of disability

A small number of women identified themselves as having more than one type of disability (n = 99), with nine having three or more disabilities. Most of this multiply-disabled group had a mental health problem as well as another disability (70%) and of those with a mental health problem, most had a physical condition or illness (75%) (data not shown).

Women in this group were significantly less likely to have a partner (66% vs. 87%) and more likely to deliver a preterm (19% vs. 7%) or LBW baby (14% vs. 5%) (Table 4). They were also significantly more likely to have 10 or more antenatal checks (37% vs. 21%) and 4 or more pregnancy scans (50% vs. 32%) (Table 5). They were also more likely to be critical about communication with staff during their pregnancy.

For labour and birth care, the women in this group were significantly more likely to have met the staff before (42% vs. 23%) and less likely to have a choice of place of birth (59% vs. 80%) (Table 6). Perceptions at this time were less likely to be positive, with significantly fewer women feeling they always had confidence and trust in the staff and that communication about their care was always effective. This was reflected in the significantly lower proportion rating labour and birth care as good or better (80% vs. 94%). This group of disabled women were significantly more likely to stay in hospital for at least 3 days (47% vs. 29%) (Table 7) and as with several of the other disabled groups, were significantly less likely to breastfeed and wished they had seen a midwife more. There were no significant differences in perceptions of communication or information giving postnatally, although they were significantly less likely to rate postnatal care overall as good or better (78% vs. 89%) in comparison with non-disabled women. Adjustment did not alter these findings.

## Discussion

The study findings across a range of disabilities present up to date information about disabled women’s use of maternity services and their views of the care provided. Overall, women with disabilities were as likely to access healthcare early on in their pregnancy as other women, but were likely to have more antenatal checks and ultrasound scans, to be delivered by caesarean section, to have longer stays in hospital and were less likely to breastfeed. Although the absolute differences were quite small, in general women with disabilities were more likely to be critical about some aspects of their care, particularly those relating to communication and support. While mothers with disability were more likely to be among those with higher numbers of postnatal midwife visits, a greater proportion would have liked more contact still in comparison with the non-disabled women. However, the women identifying as having longstanding conditions and illnesses were not a homogenous group and the comparisons of the characteristics and experience of each of the different groups with the non-disabled women illustrate this point.

Women with a physical disability, a mental health disability and those who had more than one disability all accessed antenatal services more; those who were physically disabled had less choice about place of birth and were less involved in decisions about their labour and birth care; women in all the different groups, except those with mental health problems, were more likely to have previously met the staff caring for them during labour and birth and women with learning disabilities were more critical about support and communication with staff both antenatally and intrapartum.

The findings presented reflect the different experiences of the diverse groups of women with disabilities and the way in which maternity care has to some extent begun to match needs, although there is evidence of some gaps, particularly in the relationships which women need to develop with the staff caring for them at this important time in their lives. This point is relevant in the context of both short and longer term continuity of care. Several groups stand out: women with mental health problems, those with learning disabilities and women who had more than one type of disability. Women with more than one identified disability or mental health problems were least likely to report a positive experience of pregnancy and birth. The differences were quite pronounced for some aspects of care such as communication, involvement in decisions, particularly in relation to the antenatal and labour and birth phases of care. Women with mental health problems are a heterogeneous group and training of healthcare professionals on how to communicate effectively and provide good quality care for this group is critical in terms of addressing their specific needs. However, such workers may be anxious about providing adequate care for this group of women who may in regular contact with other NHS services [22,23].

In interpreting the findings it must be held in mind that the women with learning or multiple disabilities were the smaller groups in the study. Learning disabled women may experience simultaneous discrimination and disadvantage while having additional support needs in terms of their sexuality and motherhood [24]. However, there is a growing interest in the health and welfare of women with learning disabilities, as evidenced in the literature [25]. In the present study it was clear that the needs of, for example, those women with physical disabilities were in many respects being better addressed, with greater use of services and less critical views expressed about care, particularly during pregnancy and labour and birth.

Findings from a study in Norway, involving 21 women with physical disabilities, addressed the social barriers that faced such women, suggesting that negative attitudes and limited knowledge and experience among health care professionals prevent women from receiving the perinatal care to which they are entitled [26]. Women with disabilities were slightly older and less likely to have partners. Similar findings have also been described in a review [15]. Increased risks of instrumental delivery and adverse pregnancy outcomes have been reported in women with physical disabilities and longstanding conditions [15,27,28], a finding consistent with the data in the present study relating to a higher incidence of LBW and preterm birth.

Limitations of this descriptive study which relied on self-report, include the survey response of 52% and that no data were collected on socioeconomic status or educational attainment. However, the response rate was similar to other surveys [19] and while fewer younger women participated, the sample was largely representative of the population of women giving birth in England in 2010. While women with disabilities may have been less likely to take part, nevertheless a substantial number self-identified with a disability or disabling condition. This enabled a comparison between the experience of women with disabilities and non-disabled mothers, but also some exploration of differences in care and perceptions of women with different types of disability. The numbers were not sufficient to detect more subtle differences between different types of disability, but were large enough to present the differences explored with fairly broad categories. The findings of this study were robust and the findings were largely maintained after adjustment for confounding factors.

The large scale nature of this national study and the many women responding has allowed us to describe some of the issues that smaller scale studies cannot address. While some research on disability may be better carried out by interviewing women and hearing their views directly [29], it has also been argued that in anonymous surveys women may be more frank in describing their experience of care [30].

Services providing maternity care may not be equipped to deal with the needs of women with disabilities, both practically and in organisational terms [31]. Transport, access, the design of buildings and facilities, appropriate equipment and the attitudes of staff towards disability are real issues for disabled women that were not directly addressed in this study [15], but which are likely to have been reflected in the views reported. Care may be provided by a range of different providers and not co-located so that women may experience care that is fragmented and difficult to access. Many women with disabilities have pre-existing medical conditions and are also likely to experience complications and health problems arising from the pregnancy which have a potential impact on their health and wellbeing at this time [15]. All of these aspects of maternity care for disabled women are worthy of further research. The findings from this study may enable maternity service providers to more satisfactorily pinpoint the required actions in caring for pregnant women with disability. Further research should target the experience, use of services and needs of women with different and multiple disabilities from diverse groups, using qualitative as well as quantitative methodologies. It could also include research on the role of partners and level of engagement. Audit and review of maternity services by healthcare providers should include a focus on the needs of women with disabilities represented by the different groups in this study.

## Conclusion

Women with disability have been to some extent an invisible population within the broader maternity population. It is anticipated that this study will provide evidence to support care improvements. The findings will contribute to maternity services more satisfactorily pinpointing the required actions in caring for pregnant women with different types of disability and in training staff to support women with a wide range of conditions.

We believe this study is the first to compare the use of maternity services among disabled women in the UK. We have described disabled women’s experiences of care during pregnancy, childbirth and following birth. Evidence from the survey largely indicates that in comparison with non- disabled women, those with disabilities are in general cared for similarly during pregnancy, birth and postnatally, but like all women, would prefer to have individualised care [32-34]. However, some differences were evident, with disabled women using the services more intensively and some gaps in provision were highlighted where needs could be better addressed. The implications for practice include taking the opportunities for training staff and improving aspects of maternity care for disabled women, namely in support and communication and infant feeding. This could contribute to improved outcomes for both women and babies and which could facilitate greater empowerment of the women receiving care.

## Competing interests

The authors declare they have no competing interests.

## Authors’ contributions

The authors listed have all contributed to this paper: MR designed the analysis plan; MR and RM drafted the main manuscript; HG carried out the statistical analyses; MR, RM, HG and RG contributed to the interpretation of findings and drafting the manuscript. All authors have read and approved the final manuscript.

## Pre-publication history

The pre-publication history for this paper can be accessed here:

http://www.biomedcentral.com/1471-2393/13/174/prepub
